# Nanopriming with zinc oxide: a novel approach to enhance germination and antioxidant systems in amaranth

**DOI:** 10.3389/fpls.2025.1599192

**Published:** 2025-06-25

**Authors:** Addisie Geremew, Leandrea Stovall, Selamawit Woldesenbet, Xingmao Ma, Laura Carson

**Affiliations:** ^1^ Cooperative Agricultural Research Center, College of Agriculture, Food and Natural Resources, Prairie View A&M University, Prairie View, TX, United States; ^2^ Zachry Department of Civil and Environmental Engineering, Texas A&M University, College Station, TX, United States

**Keywords:** amaranth, germination, antioxidant, zinc oxide nanoparticles, phenols, flavonoid

## Abstract

Germination is a complex physiological and biochemical process influenced by various factors, including metabolic activation and antioxidant defense mechanisms. This study investigated the effects of zinc oxide nanoparticles (ZnO NPs) of different sizes (ZnO_10_ and ZnO_35_) as seed priming agents on the germination, biochemical traits, and antioxidative systems of *Amaranthus tricolor* seeds. ZnO NPs were characterized by UV-Vis maximum peaks at 352 nm and 364 nm and average sizes of 10.0 nm and 35.2 nm for ZnO_10_ and ZnO_35_, respectively. Additionally, zeta potential indicated high stability, while transmission electron microscopy confirmed spherical morphology, energy dispersive X-ray showed high purity, and X-ray diffraction peaks indicated crystallinity. Germination percentage (GP) and germination rate (GR) were significantly improved by ZnO NP treatments, particularly at 400 mg/L, with ZnO_10_-primed seeds achieving 100% GP compared to 91.5% in ZnO_35_-primed seeds. Additionally, seedling vigor indices followed a similar trend, with ZnO_10_-primed seeds showing the highest vigor (2380) compared to ZnO_35_-primed seeds (1793.4). ZnO NPs significantly enhanced water uptake, with ZnO_10_ NPs demonstrating superior absorption at increasing concentrations, reaching a maximum of 93.6% at 400 mg/L. The α-amylase activity was also significantly higher in ZnO_10_-primed seeds (1.9 mg/g) than ZnO_35_-primed seeds (0.81 mg/g) at 400 mg/L suggesting enhanced enzymatic activation and metabolic efficiency. Antioxidant enzyme activities, including superoxide dismutase, catalase, peroxidase, ascorbate peroxidase, and glutathione peroxidase, were significantly enhanced in ZnO NP-primed seedlings, indicating improved oxidative stress management. Furthermore, lipid peroxidation, measured as malondialdehyde content, was significantly reduced, with ZnO_10_ NPs demonstrating an 89.3% reduction at 400 mg/L. The non-enzymatic antioxidant response was also enhanced, with total phenolic content and total flavonoid content significantly increased in ZnO NP-treated seedlings. The findings show that smaller-sized ZnO_10_ NPs enhance seed germination, biochemical activation, and antioxidative defense, improving seedling establishment. The high surface area of NPs enhances seed interaction and water uptake, and stimulates enzymatic activities, ultimately improving metabolic activation and protection against oxidative stress. ZnO NPs demonstrate strong potential as effective priming agents for *A. tricolor*.

## 1 Introduction

Sustainable agricultural practices aim to meet the rising global demand for crop production by improving seed germination and emergence—critical stages for successful crop development. Seeds play a vital role in agriculture, and effective management of seed inputs can significantly enhance food security. Nonetheless, stored seeds often encounter issues such as deterioration and oxidative damage, which can compromise their viability and, as a result, affect vigor and seedling establishment, ultimately impacting overall productivity (Butler et al., 2009; Alahakoon et al., 2021; Adhikary et al., 2022). In contrast, fast and consistent seed germination, along with uniform seedling development is essential for successful crop establishment to ensure economic viability and efficient use of production resources (Itroutwar et al., 2020; El-Badri et al., 2021; Sharma et al., 2021; García-Locascio et al., 2024). To address this demand, the advancement and utilization of seed treatment methods and agents that trigger biochemical and metabolic processes in seeds to enhance germination are crucial. Techniques aimed at increasing seed coat permeability to water and oxygen—such as scarification, seed coat removal, and seed nicking—have been explored with varying success in promoting germination and seedling growth (Acharya et al., 2020). However, these methods have shown limited effectiveness for smaller seeds (Fenner and Thompson, 2005). Notably, seed priming presents a promising alternative to overcome these challenges (Chatterjee et al., 2018; Waqas et al., 2019).

Seed priming is a widely applicable and efficient technique that improves key seed quality attributes, including germination speed, vigor, uniform emergence, and strong seedling growth. These enhancements contribute to increased crop productivity and greater resilience to environmental stresses (Srivastava et al., 2014; Chatterjee et al., 2018; Waqas et al., 2019; El-Badri et al., 2021; Zhou et al., 2022; Mazhar et al., 2022). Moreover, seed priming increases the activity of key enzymes such as amylases, proteases, and lipases, which are crucial for embryo growth and development (Acharya et al., 2020). Various natural and synthetic priming methods have been explored, including hydropriming (water), osmopriming (polyethylene glycol and inorganic salts), hormonal priming, nutrient priming and nanopriming (Paparella et al., 2015; Mahakham et al., 2017; Itroutwar et al., 2020; do Espirito Santo Pereira et al., 2021; Shah et al., 2021; Sharma et al., 2021; Mazhar et al., 2022). However, since each priming method has distinct characteristics and varying effectiveness depending on the crop species, careful optimization is required (Horii et al., 2007; Sytar et al., 2019).

More recently, nanopriming has emerged as a promising and effective approach to enhance seed pre-germination metabolic activities and strengthen plant resistance to various stresses (Rai-Kalal and Jajoo, 2021; Naseer et al., 2023; Khan et al., 2022). Nanoparticles (NPs), known for their small size, large surface area, and controlled release properties, have been utilized as priming agents. These unique characteristics facilitate rapid absorption, activating seed metabolism, accelerating germination, and promoting plant growth, crop protection, and overall yield improvement (Mahakham et al., 2017; Itroutwar et al., 2020; Shah et al., 2021; Mazhar et al., 2022). Several metal-based NPs such as silver nanoparticles, zinc oxide and iron oxide have been used as nanopriming agents in many crops to improve antioxidant system, increase seed vigor, enhance expression of aquaporin genes and stress mitigation (Shelar et al., 2024; do Espirito Santo Pereira et al., 2021; Nile et al., 2022).

Zinc oxide nanoparticles (ZnO NPs) have emerged as effective alternatives to conventional zinc fertilizers, enhancing zinc bioavailability in plants while also serving as efficient seed priming agents (Awan et al., 2021; Adhikary et al., 2022). Zinc is a vital micronutrient that functions as a cofactor for numerous enzymes, playing essential roles in physiological and metabolic activities such as chlorophyll and protein synthesis, growth, photosynthesis, cell elongation, pollen function, fertilization, germination, water use efficiency, membrane integrity, antioxidant defense, and disease resistance (Cakmak, 2000; Takahashi et al., 2009; Marreiro et al., 2017; Olechnowicz et al., 2018; Khanm et al., 2018; Cabot et al., 2019; Neto et al., 2020; Noohpisheh et al., 2021). Due to zinc’s vital role in human health, nanomaterial-based biofortification of crops has emerged as a promising strategy for enhancing essential nutrient content in leaves and seeds (Hussain et al., 2013; Iziy et al., 2019; Salama et al., 2019), thus, address the hidden hunger for micronutrients worldwide (Ofori et al., 2022). However, studies indicate that ZnO NPs can have both beneficial and adverse effects on germination rate, antioxidant systems, zinc accumulation, and plant growth, depending on the plant genotype, concentration, and nanoparticle size (Faizan et al., 2020; Estrada-Urbina et al., 2018; Sharma et al., 2021).

Priming small seeds is challenging due to their size, sensitivity, and susceptibility to damage during handling (Taylor et al., 1998). Uneven water absorption complicates hydration, increasing the risk of overhydration, premature germination, and viability loss (Bradford, 2002). Limited storage reserves hinder recovery from priming stress, reducing shelf life (Fenner and Thompson, 2005). Additionally, small seeds are highly sensitive to drying and storage conditions, affecting germination and vigor (Rajjou et al., 2012). Optimizing priming protocols is essential, as smaller seeds are more vulnerable to stress (Fenner and Thompson, 2005), while larger seeds benefit from greater reserves for stronger seedlings (Westoby et al., 2002).

Amaranth (*Amaranthus tricolor* L.) is a small seeded, highly nutritious leafy vegetable widely cultivated for its edible leaves and seeds. It is rich in proteins, vitamins (A, C, and folate), minerals (iron, calcium, and zinc), and bioactive compounds such as flavonoids and betalains, which contribute to its antioxidant properties (Sarker and Oba, 2020). The plant exhibits high adaptability to various environmental conditions, including drought and heat stress, making it a resilient crop for food security in arid and semi-arid regions (Rastogi and Shukla, 2013; Achigan-Dako et al., 2014). Due to its rapid growth, high yield, and nutritional benefits, amaranth is increasingly promoted as a functional food to combat micronutrient deficiencies and improve dietary diversity (Niveyro et al., 2021; Gupta and Gudu, 2022).

Considering the nutritional value of *A. tricolor* and the health benefits of zinc, this study aimed to evaluate the impact of ZnO NPs of various sizes and concentrations as a seed priming agent on the germination traits and antioxidant system of *A. tricolor* seedlings. Specifically, this research investigates how priming *A. tricolor* seeds with ZnO NPs affects their germination traits, enhances the antioxidant systems of amaranth seedlings, and increases the zinc content in amaranth seedlings. The findings from this study will provide valuable insights into the potential benefits of using ZnO NPs in agricultural practices to improve crop health and growth.

## 2 Materials and methods

### 2.1 Characterization of ZnO NPs

Two sizes of ZnO NPs were purchased from Skyspring Nanomaterials, Inc. (Houston, USA) and subjected for characterization. The UV-Vis absorption spectra of ZnO NPS were measured using a Molecular Devices ABS spectrometer over a 200–750 nm range. Particle size and the zeta potential of the samples were determined with a Litesizer 500 (Anton Paar, Austria). Scanning electron microscopy (SEM) integrated with energy-dispersive X-ray spectroscopy (EDX) (JOEL JSM-6010LA, Japan) was used to analyze the morphology of ZnO NPs and determine their elemental composition. Transmission electron microscope (TEM, JEOL-2100, Peabody, MA, USA) was employed to examine the detailed morphological characteristics of ZnO NPs at an accelerating voltage of 200 kV. The crystalline structure of ZnO NPs was analyzed using an X-ray diffractometer (XRD-7000, Shimadzu, Japan). The diffraction pattern was captured using Cu K_α_ radiation (λ = 1.541 Å) over a 2θ range of 10° to 80°.

### 2.2 Seed priming experiment

Different concentrations (50, 100, 200 and 400 mg L^-1^) of ZnO NPs and ZnSO_4_ were freshly prepared by dispersing in deionized water using ultrasonic vibration (100 w, 40 kHz) for 10 min. Distilled water was used for hydropriming. Commercial *A. tricolor* seeds (Lot #, 101294) were procured from Johnny’s Selected Seeds (Winslow, ME, USA). The seeds were sterilized by flashing with 0.1% sodium hypochlorite for 5 min and then immediately washed twice with MilliQ water. Then 1000 seeds were submersed in the corresponding treatment of 50, 100, 200 and 400 mg L^-1^ of the nanosuspensions and ZnSO_4_ of 50 mL each and constantly agitated by shaking at 160 rpm for 12 h at room temperature (Rai-Kalal and Jajoo, 2021). The seeds were then dried to restore their original moisture content following Rawat et al. (2018). After drying, the seeds were placed in polyethylene bags and stored at room temperature pending germination test and further evaluations.

### 2.3 Seed germination experiment

The ZnO NPs primed seeds were used for further germination tests and impact on seedlings enzymatic and non-enzymatic antioxidant activities. Healthy dried *A. tricolor* primed seeds were placed in Petri dishes (30 seeds per dish) bottomed by filter paper and re-hydrated with 5 mL of distilled water. Each priming condition for the respective priming materials (ZnO_10_ and ZnO_35_ NPs and distilled water) contained 4 replicates in a completely randomized design. Subsequently, Petri dishes were kept in an incubator under dark condition at 27 °C for 48 h and later transferred to light and temperature regulated growing bench. The germinated seeds were monitored daily for 6 days.

The germination traits of *A. tricolor* seeds such as germination percentage (El-Beltagi et al., 2022), mean germination time, MGT (Ellis and Roberts, 1981), germination energy, GE (Ullah et al., 2022) and mean germination rate, MGR (Alam et al., 2021) were determined. On the seventh day, 10 seedlings were randomly selected from each Petri dish to measure shoot and root length (Abou-Zeid and Mohamed, 2018). Using shoot and root lengths, vigor index was computed following Kataria et al. (2015).

### 2.4 Seed water uptake

The water uptake (WU) by *A. tricolor* seeds through the imbibition process was measured using 1000 seeds in triplicate for each treatment as described by Mazhar et al. (2022). The seeds were weighed and placed on water-saturated cotton in a Petri dish and incubated at 25°C, in 12 h intervals. All seeds were collected, blotted to eliminate excess moisture, and then weighed. Changes in weight resulting from imbibition process were computed as the water absorbed per unit of seed dry weight (Equation 1) as follow:


(1) WU=(Fresh weight of seed−Dry weight of seed)×100

### 2.5 α-Amylase activity and total soluble sugar content

To assess starch metabolism in germinated *A. tricolor* seeds, α-amylase activity was measured using a modified 3,5-dinitrosalicylic acid method (Kishorekumar et al., 2007). α-amylase was extracted from minced germinated seeds using ice-cold distilled water. Its absorbance at 540 nm was measured with a spectrophotometer, and then the α-amylase activity was calculated using a glucose standard curve. For total soluble sugar (TSS) quantification, 0.2 g of pulverized seeds were extracted with 95% ethanol, centrifuged at 5000 x g for 10 min, and the supernatant was further processed with 70% ethanol. The supernatant was then reacted with Antron reagent and heated at 100°C for 10 minutes, with absorbance recorded at 625 nm following Irigoyen et al. (1992) by using glucose for calibration.

### 2.6 Determination of antioxidant enzymes activity

A total of 1.0 g of fresh leaf samples from each treatment group was homogenized in 2.0 mL of phosphate buffer (PB) with a pH of 7.2. The resulting homogenate was then centrifuged at 10,000 rpm for 10 min. ​The supernatant obtained from this process was utilized to evaluate various stress-responsive enzymatic activities. Superoxide dismutase (SOD) activity was measured using the Cayman SOD Assay Kit (706002, Cayman Chemical, Ann Arbor, Michigan, USA). The absorbance was read at 450 nm using Spectra Max^®^ PLUS 384 plate reader. Catalase (CAT) activity in units per gram of total proteins (U TP^−1^) was assayed by measuring the reduction of H_2_O_2_ at 240 nm (Dhindsa and Matowe, 1981). Peroxidase (POD) activity was analyzed by monitoring guaiacol oxidation at 470 nm (Yue et al., 2022). Ascorbate peroxidase (APX) activity was measured by the decrease in ascorbate absorbance at 290 nm following Nakano and Asada, 1981. Furthermore, glutathione peroxidase (GPX) activity was determined using the method developed by Sattar et al. (2024), with absorbance readings at 412 nm.

### 2.7 Malondialdehyde content

MDA content in fresh seedlings was determined using a slight modification of 2-thiobarbituric acid (TBA) colorimetry method detailed by Fathi et al. (2023). Briefly, 0.1 g fresh samples were homogenized with 2 mL of phosphate-buffered saline (PBS; 50 mM, pH 7.8) and then centrifuged at 400 (r/min) for 10 min. Subsequently, 1 mL aliquot of the supernatant was combined with 1 mL of 0.5% solution of TBA dissolved in a 5% trichloroacetic acid solution. The solution was incubated for 10 min a boiling water bath and centrifuged at 10,000 x g for 10 min at 4°C. The absorbance of the resultant solution was measured at 532 nm and 600 nm and then MDA content was quantified in each sample.

### 2.8 Determination of non-enzymatic antioxidants

The total phenolic and flavonoid contents were determined using the methanol extract from the *A. tricolor* seedlings. For this, 500 mg of powdered plant material was mixed with 10 mL of 80% (v/v) aqueous methanol and shaken for 24 h at room temperature. The mixtures were then centrifuged at 10,000 rpm for 15 min. The supernatant was collected and stored at −70°C until analysis. ​To determine total phenolic content, the Folin–Ciocâlteu reagent was used, following Makkar et al. (1993). ​In this method, 250 μL of the methanol extract was combined with 1750 μL of distilled water and 100 μL of the Folin–Ciocâlteu reagent. After a 10-min incubation, 20 mL of 20% Na_2_CO_3_ solution was added. The samples were kept in the dark at room temperature for 2 h, after which the absorbance was measured at 720 nm using a UV-Vis spectrophotometer (SpectraMax^®^ PLUS 384). A standard curve was created using gallic acid at concentrations of 50, 100, 200, 300, 400, 500, 600 and 700 μg/mL.

The total flavonoid content (TFC) in *A. tricolor* seedling samples was quantified using the AlCl_3_ colorimetric technique as described by Chang et al. (2002). Specifically, 100 μL of CH_3_CO_2_K, 100 μL of AlCl_3_, and 2.8 mL of distilled water were mixed with 0.5 mL of the methanol extract. The mixtures were allowed to sit at room temperature for 30 min. Absorbance was then measured at 415 nm using a UV-Vis spectrophotometer (SpectraMax^®^ PLUS 384). Quercetin was diluted in methanol at concentrations ranging from 10 to 140 μg/mL to create the standard curve and determine the total TFC as quercetin equivalents (mg QE g^-1^ dry sample).

### 2.9 Zinc profiling of amaranth seedlings

Seedlings of *A. tricolor* were dried at 70°C for 24 h in an oven, and ground into fine powder in triplicate using mortar and pestle. About 250 mg of powdered samples from each treatment were mixed in 2 mL H_2_O_2_ (30% v/v) and 7.0 mL HNO_3_ (65% v/v) in microwave vessel and digested using a high-pressure microwave system (Milestone Ethos UP 1600, Sorisole, Italy). After the samples cooled to room temperature, the digested solutions were filtered through a 0.2-μm nylon membrane pending analyses. The concentration of Zn in each sample was analyzed using Inductive Coupled Plasma Optical Emission Spectrometer (ICP-OES, Agilent ICP-5100) integrated with Agilent SP4 autosampler at high spectral signals of wavelength of 213.86 nm.

### 2.10 Statistical analysis

The data obtained on germination traits and biochemical parameters were subjected to statistical analysis using one-way analysis of variance (ANOVA) using the different concentrations and the size of NPS (treatments) as the independent variable using JMP software (JMP pro14) and the mean values were compared using Tukey’s test (significance level 5%) for the different concentration levels of ZnO NPs. On the other hand, mean comparisons for the two sizes of NPs at a given concentration were performed using Student’s *t*-test at 5% probability level (*p* ≤ 0.05). The results are expressed as means ± standard error of the mean. The experiment was designed in a complete randomized design with triplicates per treatment.

## 3 Results and discussion

### 3.1 ZnO NPs characteristics

**Figure 1:**
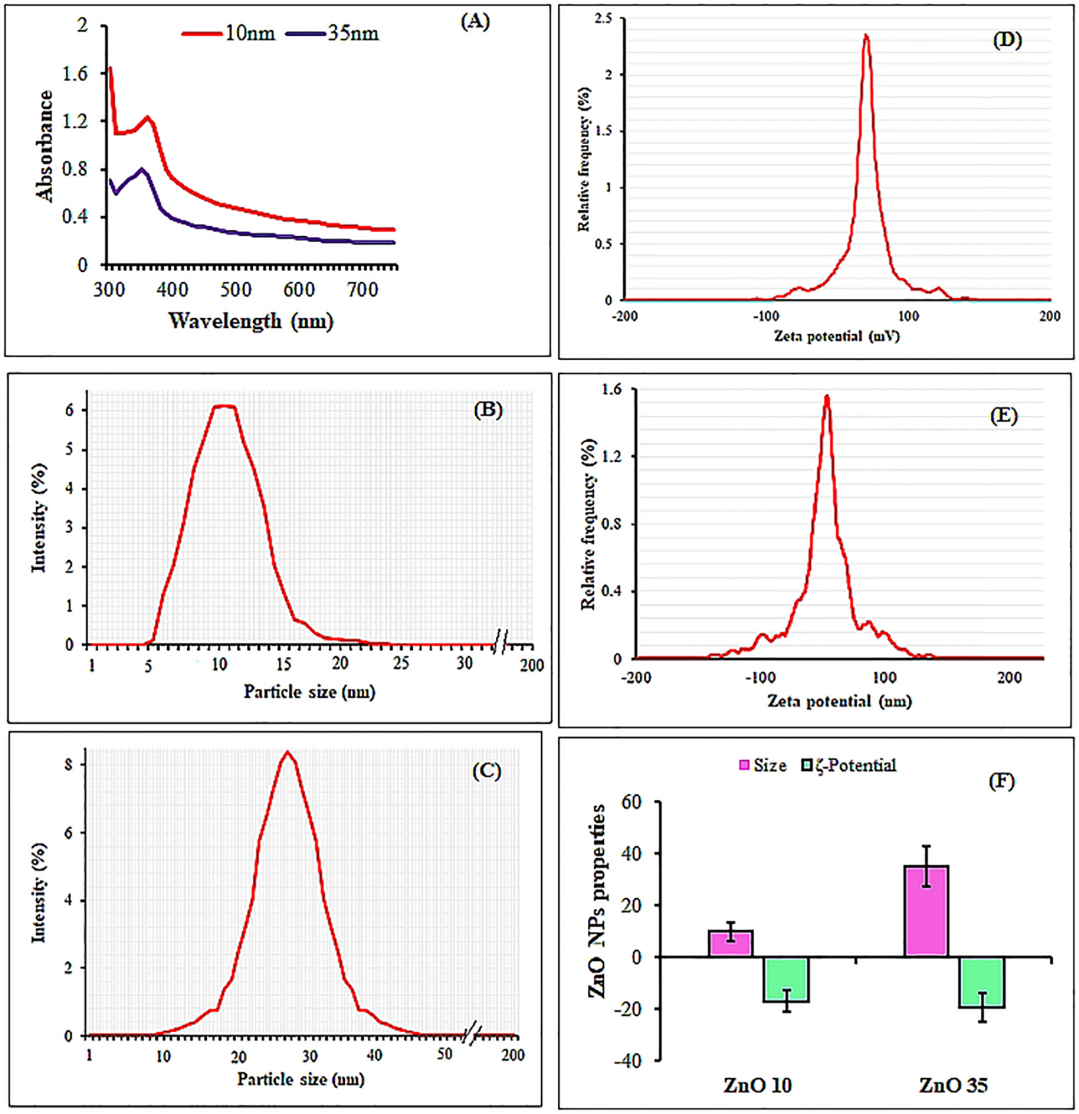
UV-Vis spectra of **(A)**, particle size distribution **(B, C)** and zeta potential **(D, E)** from DLS analysis of ZnO_10_ and ZnO_35_ nanoparticles, respectively. Also, **(F)** summarizes the average particle size and zeta potentials of ZnO_10_ and ZnO_35_ nanoparticles.

**Figure 2:**
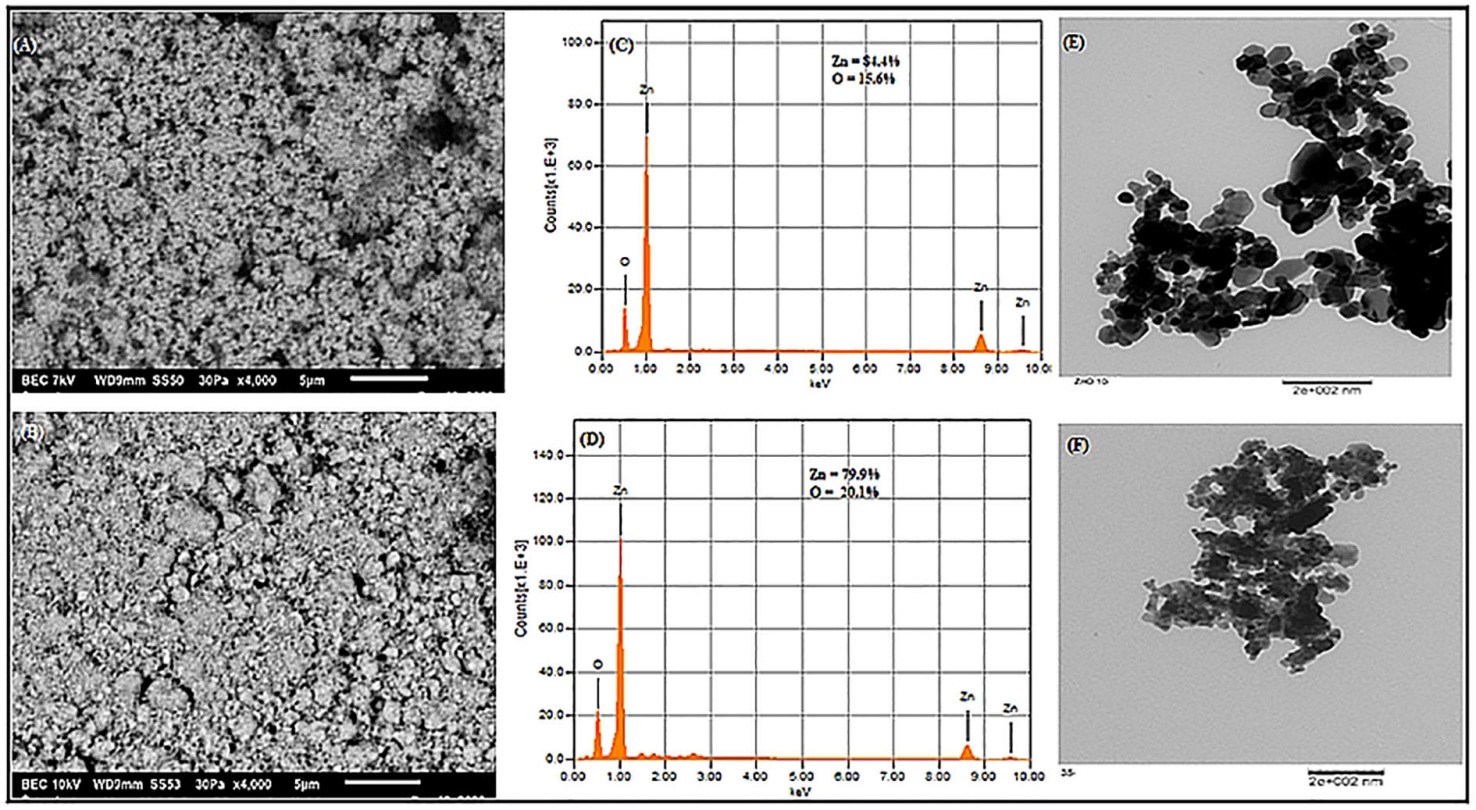
SEM images **(A, B)**, elemental composition from EDS analysis **(C, D)** and TEM images **(E, F)** of ZnO_10_ and ZnO_35_ nanoparticles.

**Figure 3:**
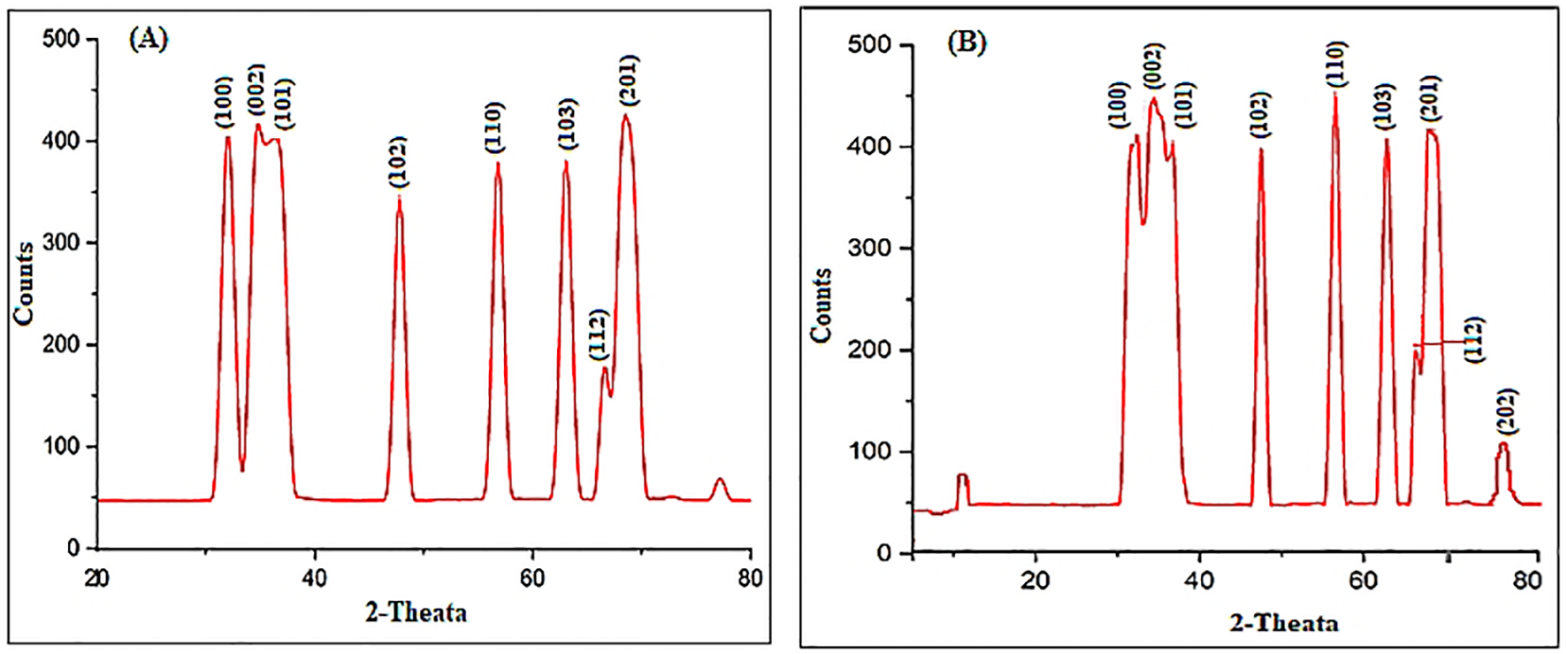
XRD patterns of ZnO_10_ **(A)** and ZnO_35_ **(B)** nanoparticles.

The UV-Vis spectral analysis displayed characteristic peaks at 352 and 364 nm, indicating the presence of ZnO NPs (**Figure 1A**). A particle size analyzer was employed to determine the average sizes of the synthesized ZnO NPs, revealing measurements of 10.0 nm and 35.2 nm (**Figures 1B, C, F**). Zeta potential analysis revealed that ZnO10 and ZnO35 NPs had values of -16.8 ± 3.2 mV and -19.3 ± 4.1 mV (**Figures 1D, E**), respectively, indicating their high stability. The high stability of the ZnO NPs in colloidal suspension is supported by zeta potential values between +30 and −30 mV, which indicate stability and high charge (Afzal et al., 2021; Khepar et al., 2023a). SEM micrographs of ZnO_10_ and ZnO_35_ revealed an aggregated particle pattern (**Figures 2A, B**). Elemental composition analysis using EDX indicated 84.4% Zn and 15.6% O for ZnO_10_, and 79.9% Zn and 20.1% O for ZnO_35_ (**Figures 2C, D**), confirming the nanoparticles’ purity (Geremew et al., 2023; Khepar et al., 2023b). Furthermore, TEM analysis confirmed the ZnO_10_ and ZnO_35_ nanoparticles’ spherical morphology with slight difference to DLS analysis with an average size of 10.9 nm and 36.2 nm, respectively (**Figures 2E, F**). Sharp peaks in XRD reflected the crystallinity of ZnO_10_ NPs, with Bragg’s reflection peaks at 2θ of 32.03°, 34.8°, 36.45°, 47.77°, 56.82°, 63.06°, 66.27° and 68.5°, corresponding to the planes (100), (002), (101), (102), (110), (103), (112) and (201) (**Figure 3A**). The peaks were matched with the ICDD card number 01-079–0207 as reported by Jayachandran et al. (2021). For ZnO35, all diffraction peaks at 2θ of 31.6°, 33.92°, 36.7°, 47.47°, 56.56°, 62.83°, 66.36°, 68.03°, and 72.05° are indexed according to the hexagonal phase of the ZnO wurtzite crystal structure with main (100), (002), (101), (102), (110), (103), (112), and smaller (201) and (202) crystal planes (**Figure 3B**). These values for ZnO35 align well with the standard JCPDS card number 04-003-2106, confirming the particle purity phase (Babayevska et al., 2022).

**Figure 4:**
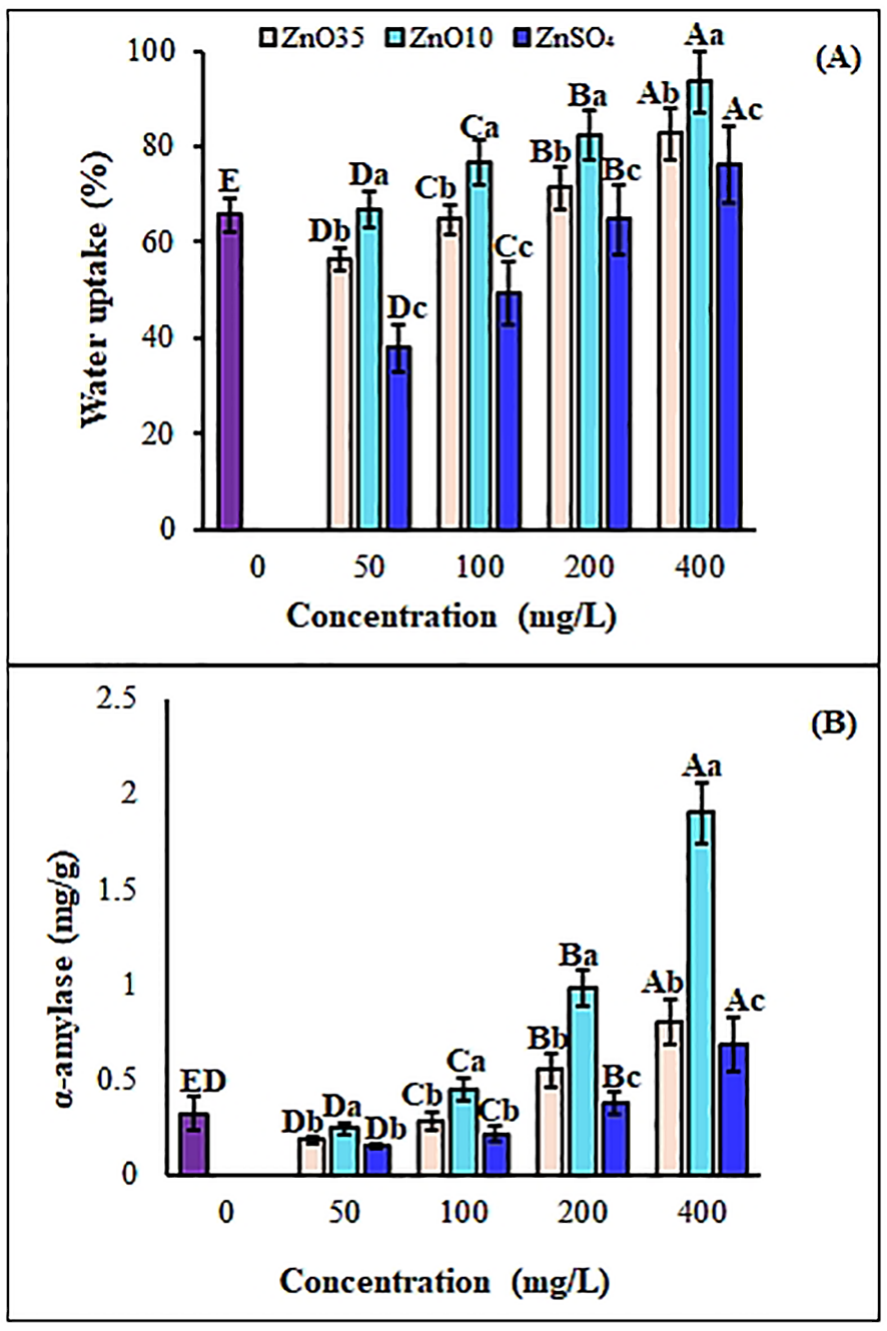
Water uptake **(A)** and alpha-amylase **(B)** of *A*. *tricolor* seeds treated with different concentrations and sizes of ZnO NPs and ZnSO_4_. Different lower-case letters denote significant differences (p< 0.05) between ZnO NPs size and ZnSO_4_ at a particular concentration. Whereas uppercase letters denote significant differences across different concentrations for a particular NP size. The purple bar shows control.

### 3.2 Effects of ZnO NPs on amaranths seed germination traits

Germination percentage (GP) of *A. tricolor* seeds primed with ZnO_10_ NPs significantly increased with dosage, reaching 72.6%, 76.3%, 87.6%, and 100% at 50, 100, 200, and 400 mg/L, respectively, compared to the control (71.3%) and ZnO_35_ NPs (50 mg/L–65.2%, 100 mg/L–72.4%, 200 mg/L–82.8%, 400 mg/L–91.5%) (p< 0.05, **Table 1**). A significant difference in GP was observed between ZnO_10_ and ZnO_35_ nano-primed seeds at 200 and 400 mg/L with the highest GP of 100% and 91.5%, correspondingly. However, the GP in seeds primed with ZnO_35_ NPs at lower concentration (50 mg/L) showed a lower efficacy in improving seed germination than that of the control significantly. Additionally, nanoprimed seeds showed a significantly higher GP than the bulk treated (ZnSO_4_) seeds with increasing concentration. At higher concentration priming amaranths seeds using ZnSO_4_ demonstrated lower percent germination and germination rate than the nanoprimed and unprimed seeds. The germination rate (GR) of *A. tricolor* seeds increased significantly with ZnO NP concentrations (50–400 mg/L) compared to the control (p< 0.05), while mean germination time showed the opposite trend (**Table 1**). The GR of ZnO_10_ and ZnO_35_ NPs treated *A. tricolor* seeds were significantly higher than unprimed seeds at 200 and 400 mg/L in the sixth days, compared to the lowest GR at 50 mg/L. Germination energy was found significantly increased in a dose dependent manner. ANOVA revealed significant differences in shoot and root lengths among seedlings from ZnO_10_ and ZnO_35_-NPs-primed seeds at 200 and 400 mg/L compared to unprimed seeds and ZnSO_4_ primed seeds (**Table 1**).

**Table 1:** Effect of ZnO NPs and ZnSO_4_ priming treatments on germination and growth parameters of *A. tricolor*.

Nanoparticles	Concentration (mg/L)	GP (%)	MGT (days)	GR	GE	RL (cm)	SL (cm)	VI
Control (water)	0	71.3^Ca^	5.63^DB^	0.18^B^	61.5^EC^	6.3^BD^	5.4^D^	834.21^E^
ZnO_10_	50	72.6^Ca^	6.87^Da^	0.15^Cb^	52.3^Db^	5.3^Da^	7.5^Ca^	929.3^DEa^
	100	76.3^Ca^	4.21^Cb^	0.24^Ca^	69.4^Ca^	6.4^Ca^	8.5^Ca^	1136.9^Ca^
	200	87.6^Ba^	3.33^Bc^	0.30^Aa^	82.5^Ba^	7.5^Ba^	10.5^Ba^	1576.8^Ba^
	400	100^Aa^	2.58^Ac^	0.39^Aa^	91.2^Aa^	10.3^Aa^	13.5^Aa^	2380.0^Aa^
ZnO_35_	50	65.2^Db^	5.42^Db^	0.18^Bb^	45.3^Dc^	3.5^Db^	5.2^Db^	567.2^Dc^
	100	72.4^Ca^	4.75^Ca^	0.21^Ba^	60.8^Cb^	4.2^Cb^	7.3^Ca^	832.6^Cb^
	200	82.8^Bb^	4.08^Bb^	0.24^Bb^	71.5^Bb^	6.5^Ba^	8.5^Bb^	1242.0^Bb^
	400	91.5^Ab^	3.04^Ab^	0.33^Aa^	80.2^Ab^	9.1^Aa^	10.5^Ab^	1793.4^Ab^
ZnSO_4_	50	76.8^Dc^	4.22^Dc^	0.25^Ca^	70.3^Da^	5.2^Da^	4.8^Dc^	768.0^Db^
	100	70.4^Cc^	5.41^Ca^	0.18^Ba^	60.5^Cb^	3.7^Cb^	3.8^Cb^	528.0^Cc^
	200	58.5^Bc^	6.05^Ba^	0.16^Bc^	40.6^Bc^	2.4^Bb^	2.2^Bc^	269.1^Bc^
	400	41.9^Ac^	7.01^Aa^	0.11^Ab^	32.6^Ac^	1.5^Ab^	1.0^Ac^	104.6^Ac^

Different lower-case letters denote significant differences (p< 0.05) between ZnO NPs size and ZnSO_4_ at a particular concentration. Whereas uppercase letters denote significant differences across different concentrations for a particular NP size and bulk ZnSO_4_. GP, germination percentage; MGT, mean germination time; GR, germination rate; GE, Germination energy; RL, root length; SL, shoot length; and VI, vigor index.

Interestingly, priming *A. tricolor* seeds with ZnO_10_ and ZnO_35_ NPs (50–400 mg/L) significantly enhanced seedling vigor, ranging from 929.3 to 2380 and 567.2 to 1793.4, respectively (**Table 1**). In contrast a significant decrease in seedling vigor was observed in ZnSO_4_ primed seeds with augmented concentrations. The highest seedling vigor indices were recorded for ZnO_10_ (2380) and ZnO_35_ (1793.4) NPs at 400 mg/L. Overall, nanopriming with ZnO NPs enhanced vigor for all priming treatments (P< 0.05). In agreement with our findings, recently, ZnO NPs based priming increased seed germination characteristics such as GP, GR, and vigor (Khalaki et al., 2021). Similarly, Khepar et al. (2024) have reported improved germination traits in rice seeds primed with ZnS nanoparticles. Seeds primed with ZnO NPs exhibited enhanced germination and vigorous seedling growth owing to zinc’s essential role in inducing protein and carbohydrate metabolism, breaking of dormancy, imbibition and enzyme activation, which are critical for early coleoptile and radicle development (Samad et al., 2013; Farooq et al., 2013; Shah et al., 2019; Neto et al., 2020; Rai-Kalal and Jajoo, 2021; Ozturk et al., 2006; do Espirito Santo Pereira et al., 2021; Del Buono et al., 2022). The increased GR and seedling vigor of nanoprimed seeds in this study may be attributed to enhanced α-amylase activity (discussed in the section follows), which accelerates starch hydrolysis during germination (Sharifan et al., 2020; Tondey et al., 2021; Gupta et al., 2022; Kathiravan et al., 2024).

In addition, the enhanced radicle length may be attributed to zinc’s role in modulating hormone metabolism, particularly its influence on auxin levels through the regulation of tryptophan biosynthesis as well as its essential role in biosynthesis of gibberellins (Prasad et al., 2012; Kathiravan et al., 2024). Zinc’s hormonal modulation effect is known to control the early stages of seed germination and radicle development (El-Badri et al., 2021). Also, nanopriming with ZnO NPs often improves germination traits, shoot and root lengths, and seedling vigor by facilitating seed coat penetration and increasing pore formation (Hatami, 2017; Hatami et al., 2019). Thus, these phenomena promoted oxygen transfer to seeds and water uptake potential (Afzal et al., 2021). However, it has been found that ZnO NPs can result in different effects on radicle elongation, also causing severe toxic effects (Stałanowska et al., 2023). In all measured germination traits, significant differences were observed between ZnO_10_ and ZnO_35_ NPs. Smaller NPs create more seed coat pores, enhancing water uptake and upregulating aquaporin gene expression compared to larger NPs (Tondey et al., 2021; Gupta et al., 2022; Qian et al., 2013; Kathiravan et al., 2024) and as a consequence enhance seed germination and seedling growth more effectively (Hussain et al., 2017; Afzal et al., 2021). Moreover, during early germination, NPs generate ROS (shown in next sections) as signaling molecules, facilitating reserve mobilization, cell wall loosening, endosperm weakening, improved water absorption, and cell extension, ultimately enhancing germination (Oracz and Karpinski, 2016; Mahakham et al., 2017; Del Buono et al., 2022; Itroutwar et al., 2020).

### 3.3 Biochemical parameters

To initiate germination and growth, seeds must absorb an adequate amount of water. In the present study, the percentage of water uptake was found higher in *A. tricolor* seeds when primed with ZnO_10_ and ZnO_35_ NPs as compared to the control and ZnSO_4_ (**Figure 4A**). Also, the water absorption capacity of the seeds increased with the increased concentration of ZnO NPs and ZnSO_4_ priming solution. The seed water uptake percentage of primed *A. tricolor* seeds at 50, 100, 200 and 400 mg/L of ZnO_10_ NPs dosage was 1.7%, 16.7%, 25.3%, and 42.5%, respectively which was significantly higher (p ≤ 0.05) as compared to distilled water (**Figure 2A**). The maximum water uptake achieved was 93.6% at 400 mg/L of ZnO_10_ NPs primed *A. tricolor* seeds. While the seed water uptake percentage of ZnO_35_ NP-primed seeds at 50 and 100 mg/L did not increase significantly, seeds primed with 200 and 400 mg/L showed significant increases of 8.7% and 25.7%, respectively, compared to the control. The maximum water uptake was 82.6% at 400 mg/L priming concentrations of ZnO_35_ NPs for *A. tricolor* seeds. Water uptake by ZnSO_4_ primed seeds did not show significant water uptake at 50, 100 and 200 mg/L relative to the control (water). NPs may interact with cell walls to create micropores in the seed coat, thereby enhancing water uptake and upregulating the expression of aquaporin genes (Qian et al., 2013; Kathiravan et al., 2024) and ultimately accelerating seed germination (Soni et al., 2023).

Alpha-amylase content in the seeds plays a vital role in the hydrolysis of endosperm starch to sugars for metabolism. The α-amylase activity in ZnO_35_ NPs-primed *A. tricolor* seeds at 50, 100, 200, and 400 mg/L was 0.18, 0.28, 0.55, and 0.81 mg/g, respectively, while ZnO_10_ NPs-primed seeds showed activities of 0.24, 0.44, 0.98, and 1.9 mg/g at the same concentrations (**Figure 4B**). A significant difference (p< 0.05) in α-amylase activity was observed between ZnO_35_ and ZnO_10_ NPs-primed seeds and ZnSO_4_ primed seeds at 100, 200 and 400 mg/L, as well as compared to the control (0.32 mg/g). However, no significant enhancement was noted at 50 mg/L for either nanoparticle or ZnSO_4_ treatment relative to the control. The enhanced α-amylase activity in ZnO NPs-primed seeds may result from increased water uptake during imbibition (Rai-Kalal and Jajoo, 2021), as the case in this study. Our findings align with previous studies highlighting the role of nanopriming in enhancing starch metabolism during germination, where α-amylase facilitates nutrient mobilization and carbohydrate conversion to soluble sugars, supporting germination and seedling growth (Zheng et al., 2016; Sharma et al., 2021). In this regard, zinc ions released from ZnO NPs might activate α-amylase (Rai-Kalal and Jajoo, 2021) and then augment starch hydrolysis that increase soluble sugars to fuel seedling growth (Kathiravan et al., 2024 (Choudhary et al., 2019; Veˇceˇrová et al., 2016; Del Buono et al., 2022). Additionally, it has also been confirmed that some metal-based NPs can cross the seed coat and stimulate the embryonic differentiation by inducing the enzymes that interrupt seed dormancy (García-Locascio et al., 2024).

**Figure 5:**
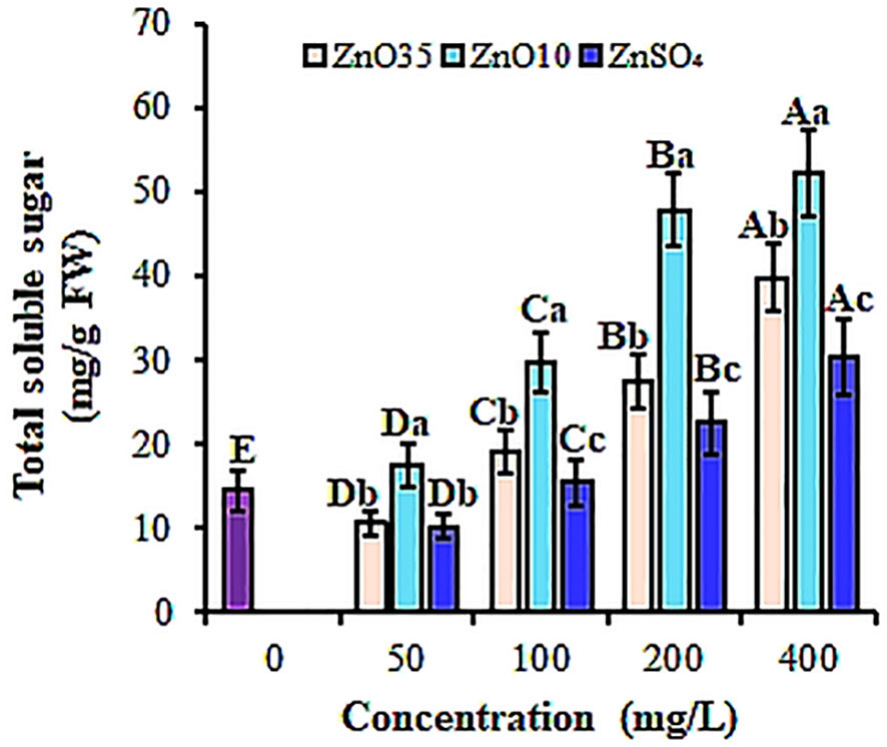
Total soluble sugar content of *A. tricolor* seeds treated with different concentrations and sizes of ZnO NPs and ZnSO_4_. Different lower-case letters denote significant differences (p< 0.05) between ZnO NPs size and ZnSO_4_ at a particular concentration. Whereas uppercase letters denote significant differences across different concentrations for a particular NP size. The purple bar shows control.

This study also showed significant stimulation (p< 0.05) in total soluble sugar (TSS) in ZnO NPs-primed *A. tricolor* seeds compared to the control and the bulk ZnSO_4_, varying with concentration (**Figure 5**). Compared to the control, both ZnO_35_ and ZnO_10_ NPs at 400 mg/L resulted in the highest TSS content of *A. tricolor* seeds by 63.6% and 72.3%, respectively. The increase in TSS could be attributed to the higher water absorption and α-amylase activity (Sharma et al., 2021). In agreement with our findings Acharya et al. (2020) and Sharma et al. (2021) found that watermelon and rice seeds treated with Ag NPs and ZnO NPs had higher soluble sugar content during germination compared to untreated seeds.

### 3.4 Seed priming with ZnO NPs modulates antioxidative systems

Antioxidative systems, including enzymatic and non-enzymatic components, are essential for neutralizing ROS and maintaining cellular homeostasis in plants (Ahammed et al., 2020; Soni et al., 2023). To counteract ROS, plants activate non-enzymatic antioxidants like phenols and flavonoids, along with enzymatic antioxidants such as catalase (CAT), peroxidase (POD), and superoxide dismutase (SOD). Antioxidant enzymes serve as positive regulators by controlling ROS production and maintaining the balance between its generation and elimination (Bailly et al., 2008; Sharma et al., 2021; Soni et al., 2023).

**Figure 6:**
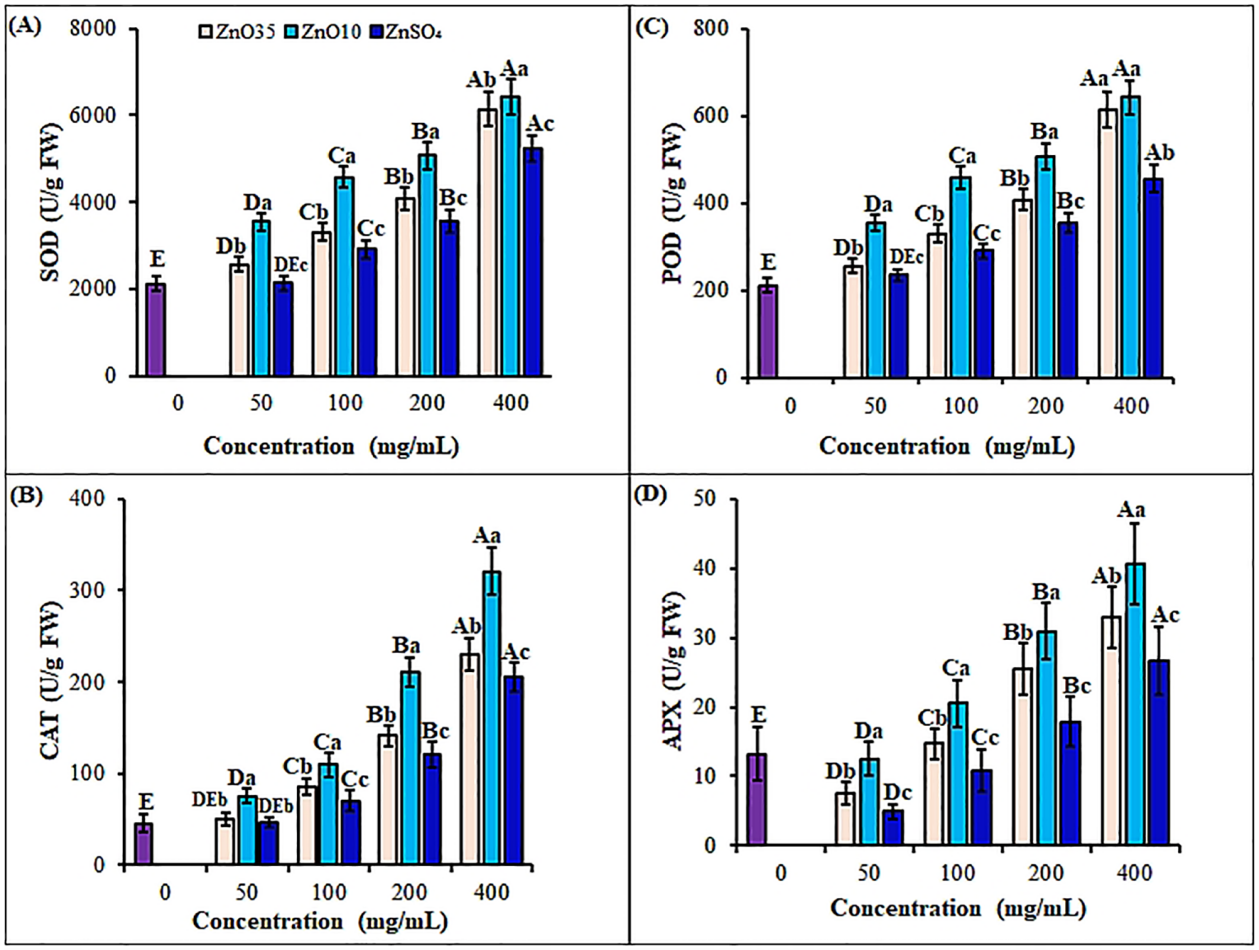
Antioxidant enzymes: SOD **(A)**, CAT **(B)**, POD **(C)** and APX **(D)** contents of *A*. *tricolor* seedlings from seeds primed with different concentrations and sizes of ZnO NPs and ZnSO_4_. Different lower-case letters denote significant differences (p< 0.05) between ZnO NPs and ZnSO_4_ size at a particular concentration. Whereas uppercase letters denote significant differences across different concentrations for a particular NP size. The purple bar shows control.

**Figure 7:**
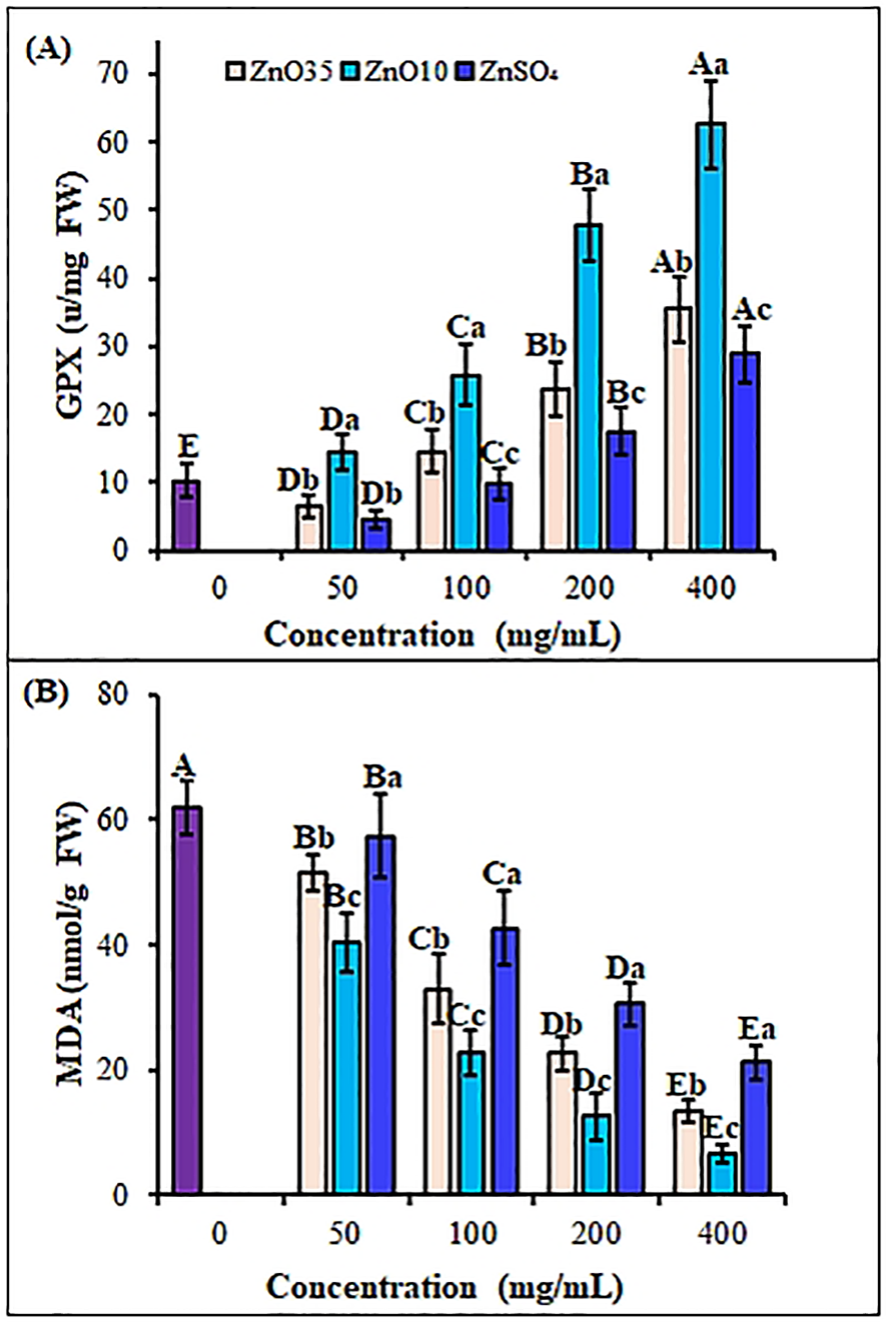
Antioxidant and reactive oxygen response enzymes GPX **(A)** and, MDA **(B)** contents, respectively of *A*. *tricolor* seedlings from seeds primed with different concentrations and sizes of ZnO NPs and ZnSO_4_. Different lower-case letters denote significant differences (p< 0.05) between ZnO NPs and ZnSO_4_ size at a particular concentration. Whereas uppercase letters denote significant differences across different concentrations for a particular NP size. The purple bar shows control.

*A. tricolor* seedlings oxidative status was investigated by exploring the effect of different size and concentrations of ZnO NPs and ZnSO_4_ as priming agents on antioxidant enzymes and non-enzymatic antioxidants during early seedling growth. Priming of *A. tricolor* seeds with ZnO NPs significantly (p< 0.05) enhanced antioxidant enzyme activities compared to the control and the bulk ZnSO_4_ (**Figures 6A-D**, **Figure 7A**). SOD activity rose under all ZnO_10_ and ZnO_35_ NPs treatments, with increases of 40.3% to 67.1% and 17.3% to 33.5%, respectively, at concentrations of 50–400 mg/L relative to the control (**Figure 6A**). CAT activity was also increased by about 10%, 15%, 23%, and 44% in seedlings treated with ZnO_35_ NPs and 16%, 24%, 46%, and 60.8% in ZnO_10_ NPs under the same concentration range (**Figure 6B**). Also, ZnO_10_ and ZnO_35_ NP priming significantly enhanced enzyme activities (P< 0.05), with POD increasing by 203.5% and 190.5% (**Figure 6C**), APX by 207.6% and 149.2% (**Figure 6D**) and, GPX by 513.7% and 248% (**Figure 7A**), respectively, in *A. tricolor* seedlings primed at a concentration of 400 mg/L compared to unprimed seedlings. While priming with 50 mg/L ZnSO_4_ did not show a significant difference in the stimulation of SOD, CAT, and POD compared to the control, it resulted in notably lower levels of SOD, CAT, POD, APX, and GPX when compared to ZnO_10_ and ZnO_35_ NPs across various concentrations. This reflects the minimal effect of the bulk ZnSO4 on antioxidant enzyme enhancement (Khepar et al., 2024). In most instances, the highest antioxidant enzyme activities were noted at maximum ZnO NPs doses, indicating their effectiveness in overcoming oxidative stress. The high SOD activity in ZnO NPs primed *A. tricolor* seedlings could be linked to augmented binding of Zn^2+^ to thiols, which induced its synthesis (Li et al., 2021). Additionally, the interconnectedness of the antioxidant enzymes activities like the SOD and its isoenzyme (Zn-SOD) is an important factor for the consistent pattern of their increase (Hajiboland, 2014; López-Vargas et al., 2020). For instance, SOD converts superoxide radicals into H_2_O_2_ and O_2_, while CAT, POD, APX, and GPX further break down H_2_O_2_ into H_2_O and O_2_, with APX specifically involved in H_2_O_2_ scavenging via the glutathione-ascorbate cycle (Soni et al., 2023; Kibinza et al., 2011; Sharma et al., 2021; Afzal et al., 2021). In agreement with our results, increase of antioxidant enzymes have also been observed with the application of ZnO NPs as priming agents in seedlings of several crops such as green gram (Kathiravan et al., 2024), rice (Mazhar et al., 2022; Sharma et al., 2021), pearl millet (Kumar et al., 2024), black gram (Banerjee et al., 2023) and wheat (Rai-Kalal and Jajoo, 2021; Wang et al., 2020) as well as different vegetables (Younes et al., 2020; Sharma et al., 2021; Salam et al., 2022; Selim et al., 2020; Ruszkiewicz et al., 2017). Studies have also shown nano-primed seeds trigger oxidative bursts during germination, fortifying antioxidant defense mechanisms and promoting enhanced seedling vigor and plant growth throughout post-priming germination stages (Chen and Arora, 2013; Shinde et al., 2020; Afzal et al., 2021; Kathiravan et al., 2024; Khepar et al., 2024). As reported for ZnS, FeS and MnS NPs primed seedlings of rice and Brassica the enhancement of antioxidant enzymes in ZnO NPs primed amaranths could linked with the upregulation of target antioxidant genes (Khepar et al., 2024) and elevated CAT and APX transcript levels (Shaw and Hossain, 2013; Sharma et al., 2012). Additionally, nanopriming facilitates the formation of nanopores in shoots, aiding water absorption and activating antioxidant mechanisms, thus enhancing seed germination and growth (Chen and Arora, 2013; Nile et al., 2022; Khepar et al., 2023a). However, priming treatments do not always boost the activity and expression of antioxidant enzymes (Goswami et al., 2013; Farooq et al., 2022) as a result of phytotoxicity, genotype difference and NPs size and concentration discrepancy.

A balance between ROS production and elimination is essential for successful seed germination and seedling development (Naseer et al., 2023). The MDA content in *A. tricolor* seedlings was significantly affected by ZnO NPs treatments. The MDA content was reduced significantly by 16.9%, 46.8%, 63.5% and 78.2% at ZnO_35_ NPs at 50, 100, 200 and 400 mg/L, respectively as compared to untreated control (**Figure 7B**). A similar pattern of MDA content reduction—34.9%, 63.4%, 79.7%, and 89.3%—was observed for the corresponding ZnO_10_ NP concentrations relative to the control. ZnO_10_ NPs and ZnO_35_ NPs exhibited a significant difference in MDA content (p< 0.05). Despite the decrease in MDA level ranging from 7.4% to 65.6% with increase in ZnSO_4_ concentration, the MDA level was significantly higher than the ZnO_10_ and ZnO_35_ NPs (p< 0.05). The reduction in MDA content and the enhanced activity of antioxidant enzymes in *A. tricolor* seedlings primed with ZnO NPs suggest a significant decrease in ROS activity. Align with these findings, numerous studies have demonstrated that NPs bolster plant antioxidant systems, mitigating oxidative damage by scavenging ROS, as evidenced by reduced MDA levels (Mishra et al., 2014; Sharma et al., 2021; Kathiravan et al., 2024).

**Figure 8:**
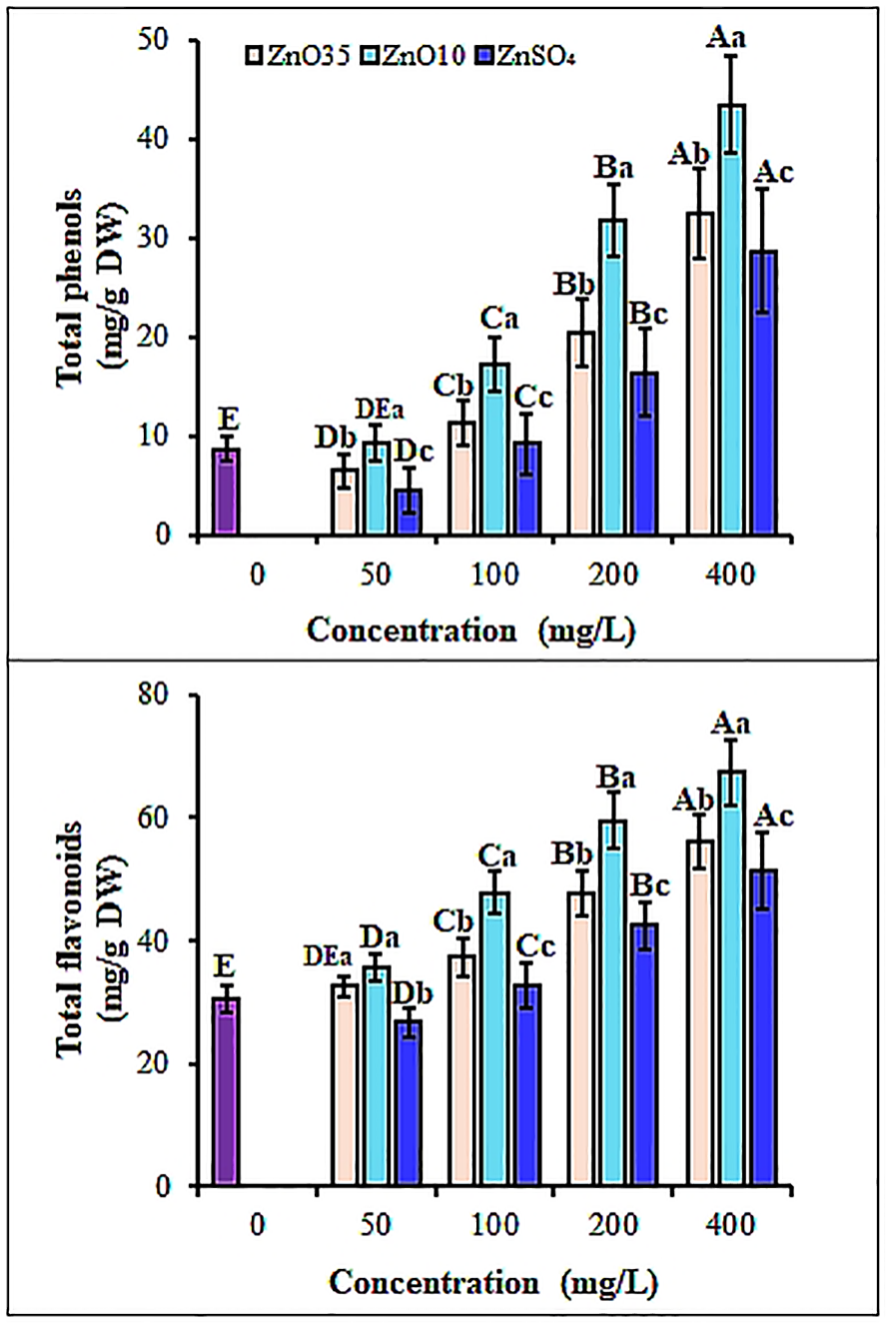
Total phenolic **(A)** and total flavonoids **(B)** content of *A*. *tricolor* seedlings from seeds primed with different concentrations and sizes of ZnO NPs and ZnSO_4_. Different lower-case letters denote significant differences (p< 0.05) between ZnO NPs and ZnSO_4_ size at a particular concentration. Whereas uppercase letters denote significant differences across different concentrations for a particular NP size. The purple bar shows control.

To overcome oxidative stress, together with enzymatic antioxidant system, plants detoxify ROS by upregulating production of nonenzymatic antioxidant phytomolecules including phenols and flavonoids (Singh et al., 2019; Rizk et al., 2025). Seedlings from both ZnO_10_ and ZnO_35_ primed *A. tricolor* seeds revealed a significant (p< 0.05) increase in total phenolic content (TPC) by 49.7% and 31% at 100 mg/L, and 79.9% and 73.2% at 400 mg/L, respectively (**Figure 8A**) compared with the control. While the ZnSO_4_ primed seedlings showed an increased TPC, overall, the magnitude was significantly lower than ZnO_10_ and ZnO35 NPs (P< 0.05). Similarly, studies indicate that plants produce phenolic compounds in response to nanoparticle exposure as a defense against oxidative stress and ROS (Acharya et al., 2020; Rai-Kalal and Jajoo, 2021; Sofo et al., 2017; Del Buono et al., 2022). Although the mechanisms underlying nanoparticle-induced phenol synthesis are largely unknown, studies indicate that ZnO NPs may influence this process through transcriptional regulation (Abbasi et al., 2019). Additionally, the result shown in **Figure 8B** depicts that with ZnO_10_ and ZnO_35_ NPs treatment, TFC increased by about 14.3% and 6.4% in 50 mg/L, 36.2% and 18.4 in 100 mg/L, 48.7% and 36.1% in 200 mg/L, 54.7% and 45.7% in 400 mg/L, correspondingly. Though the ZnSO_4_ primed seedlings showed stimulating effect on the TFC, their effect is significantly lower than ZnO10 or ZnO_35_ NPs. NPs have been reported to boost flavonoid content that can reduce lipid peroxidation and mitigate photo-oxidative damage in seedlings (Del Buono et al., 2022; López-Vargas et al., 2020; Banerjee et al., 2021). Higher phenol and flavonoid levels may be linked to their metal-chelating properties, which help limit toxic metal accumulation to optimal levels (Gulcin and Alwasel, 2022). The biostimulatory effects of ZnO NPs in plants depend on their physical properties, including size, shape, roughness, and composition (Juárez-Maldonado et al., 2021), as highlighted by the significant impact of size on flavonoid and phenolic content in this study.

### 3.5 Effect on zinc profile of A. tricolor seedlings

**Figure 9:**
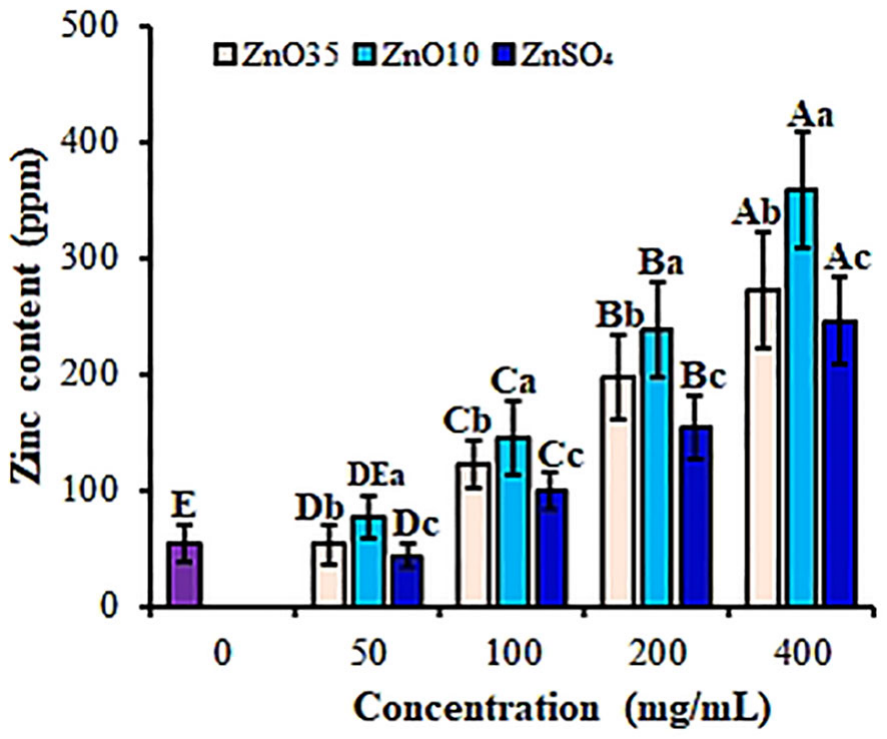
Zinc content of *A. tricolor* seedlings from seeds primed with different concentrations and sizes of ZnO NPs and ZnSO_4_. Different lower-case letters denote significant differences (p< 0.05) between ZnO NPs and ZnSO_4_ size at a particular concentration. Whereas uppercase letters denote significant differences across different concentrations for a particular NP size. The purple bar shows control.

Zinc is an essential element necessary for growth and development of plants (Pathak et al., 2012). In this study, nanopriming with ZnO_10_ and ZnO_35_ NPs led to a significant enhancement in zinc content in *A. tricolor* seedlings, as verified by ICP-OES measurements. The zinc accumulation displayed a dose-dependent trend (P< 0.05), with the highest efficacy observed at 400 mg/L (**Figure 9**). ZnO NPs significantly differ in increasing Zinc content compared to the ZnSO4 despite concentration. At this concentration, ZnO_10_ and ZnO_35_ NPs increased zinc content by 85% and 80%, respectively, compared to the control, demonstrating effective biofortification through nanoparticle priming. Consistent with these findings, earlier studies have reported that priming with ZnO NPs improves morphometric traits and elevates zinc levels in crops such as maize (Naseer et al., 2023) and wheat (Pandya et al., 2024) at concentrations of 250 and 450 mg/L, respectively. The effect could be partly explained by their small size and high surface area which facilitates better absorption and distribution of zinc within the plant (Sharma et al., 2021; Itroutwar et al., 2020). Furthermore, studies highlight significant zinc content partitioning between the shoots and roots of these crop species (Rameshraddy et al., 2018; Srivastav et al., 2021; Pandya et al., 2024). Similarly, Francisco et al. (2024) demonstrated that priming lettuce with smaller ZnO NPs resulted in a 3.2- to 12.6-fold increase in zinc concentration in leaves, further emphasizing the potential of ZnO NPs for effective biofortification. Studies have also shown that nanopriming with FeS NPs and MnS NPs demonstrated nutritional modulation by enhanced uptake of nanoforms of iron and manganese in rice (Khepar et al., 2023a). ZnO-NP priming significantly increases zinc content in edible plant parts, addressing widespread zinc deficiency in human diets and offering a sustainable, efficient approach to enhance crop nutritional quality, thereby contributing to better health outcomes (Ashwini et al., 2024; Itroutwar et al., 2020).

## 4 Conclusions

The study demonstrates that ZnO NP) are effective seed priming agents for Amaranthus tricolor, significantly enhancing germination traits, seedling vigor, and antioxidant enzyme activities. Improved water uptake, α-amylase activity, and total soluble sugar content are critical for early seedling growth, while increased antioxidant enzyme activities and reduced malondialdehyde content indicate enhanced oxidative stress resistance. ZnO NPs also boost zinc content in seedlings, highlighting their potential for biofortification. These findings suggest that ZnO nanopriming is a promising and sustainable technique to enhance seed germination, seedling growth, and overall crop vigor, offering a practical approach to improve agricultural productivity and resilience to environmental stress, thereby contributing to sustainable agriculture and food security.

## Data Availability

The original contributions presented in the study are included in the article/**Supplementary Material**. Further inquiries can be directed to the corresponding author.

## Supplementary material

Supplementary Figure 1
*A. tricolor* seedlings from ZnO NPs and ZnSO_4_ primed seeds using different concentrations.

Supplementary Figure 2 Calibration curve used to determine total phenolic content of *A. tricolor* seedlings.

Supplementary Figure 3 Calibration curve used to determine total flavonoids content of *A. tricolor* seedlings.
