# Supplementary figures and images for: Nanopriming with zinc oxide: a novel approach to enhance germination and antioxidant systems in amaranth
Source: Front Plant Sci. 2025 Jun 25;16:1599192. doi: 10.3389/fpls.2025.1599192 (PMC12237966; doi:10.3389/fpls.2025.1599192)

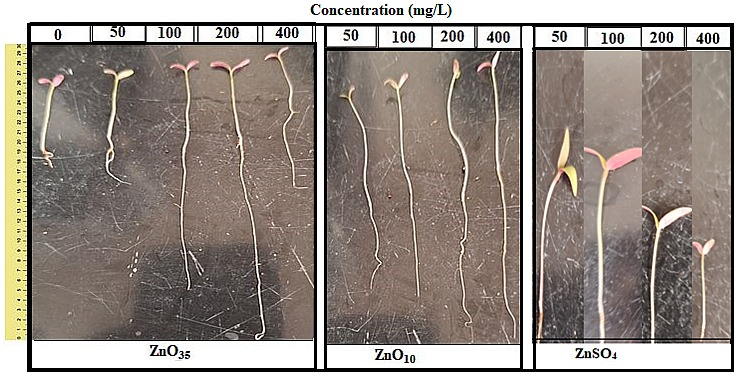

Supplement: Supplementary Figure 1 — A. tricolor seedlings from ZnO NPs and ZnSO4 primed seeds using different concentrations. [file Image1.tif]

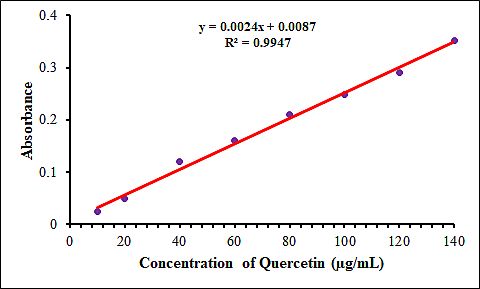

Supplement: Supplementary Figure 2 — Calibration curve used to determine total phenolic content of A. tricolor seedlings. [file Image2.jpeg]

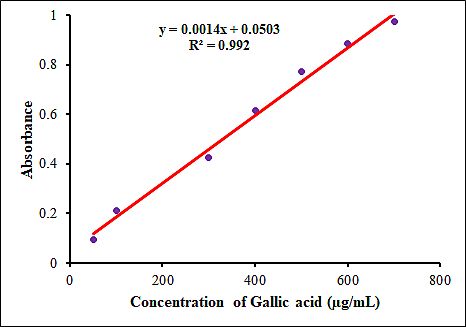

Supplement: Supplementary Figure 3 — Calibration curve used to determine total flavonoids content of A. tricolor seedlings. [file Image3.jpeg]
